# Abnormalities in Oxygen Sensing Define Early and Late Onset Preeclampsia as Distinct Pathologies

**DOI:** 10.1371/journal.pone.0013288

**Published:** 2010-10-12

**Authors:** Alessandro Rolfo, Ariel Many, Antonella Racano, Reshef Tal, Andrea Tagliaferro, Francesca Ietta, Jinxia Wang, Martin Post, Isabella Caniggia

**Affiliations:** 1 Samuel Lunenfeld Research Institute, Mount Sinai Hospital, Toronto, Ontario, Canada; 2 Department of Obstetrics and Gynecology, University of Toronto, Toronto, Ontario, Canada; 3 Department of Physiology, University of Toronto, Toronto, Ontario, Canada; 4 Department of Pediatrics, University of Toronto, Toronto, Ontario, Canada; 5 The Hospital for Sick Children, Toronto, Ontario, Canada; Institute of Zoology, Chinese Academy of Sciences, China

## Abstract

**Background:**

The pathogenesis of preeclampsia, a serious pregnancy disorder, is still elusive and its treatment empirical. Hypoxia Inducible Factor-1 (HIF-1) is crucial for placental development and early detection of aberrant regulatory mechanisms of HIF-1 could impact on the diagnosis and management of preeclampsia. HIF-1α stability is controlled by O_2_-sensing enzymes including prolyl hydroxylases (PHDs), Factor Inhibiting HIF (FIH), and E3 ligases Seven In Absentia Homologues (SIAHs). Here we investigated early- (E-PE) and late-onset (L-PE) human preeclamptic placentae and their ability to sense changes in oxygen tension occurring during normal placental development.

**Methods and Findings:**

Expression of PHD2, FIH and SIAHs were significantly down-regulated in E-PE compared to control and L-PE placentae, while HIF-1α levels were increased. PHD3 expression was increased due to decreased FIH levels as demonstrated by siRNA FIH knockdown experiments in trophoblastic JEG-3 cells. E-PE tissues had markedly diminished HIF-1α hydroxylation at proline residues 402 and 564 as assessed with monoclonal antibodies raised against hydroxylated HIF-1α P402 or P564, suggesting regulation by PHD2 and not PHD3. Culturing villous explants under varying oxygen tensions revealed that E-PE, but not L-PE, placentae were unable to regulate HIF-1α levels because PHD2, FIH and SIAHs did not sense a hypoxic environment.

**Conclusion:**

Disruption of oxygen sensing in E-PE *vs.* L-PE and control placentae is the first molecular evidence of the existence of two distinct preeclamptic diseases and the unique molecular O_2_-sensing signature of E-PE placentae may be of diagnostic value when assessing high risk pregnancies and their severity.

## Introduction

Preeclampsia is a placental disorder that affects about 5–10% of all pregnancies and clinically manifests itself in the third trimester with a wide variety of maternal symptoms, including hypertension, proteinuria, and generalized edema [1], [2]. The placenta plays a key role in the genesis of this disease as its removal at the time of delivery results in rapid resolution of the clinical symptoms. Although preeclampsia appears suddenly in the third trimester, the initial insult underlying its genesis occurs likely in the first trimester of pregnancy, at the time when trophoblast cell differentiation/invasion commences. Histomorphological studies have reported that preeclamptic pregnancies are characterized by defective remodelling of maternal spiral arteries due to poor invasion by trophoblast cells in the decidua [3]. Consequently, vessels at the maternal-placental interface remain highly resistant and utero-placental perfusion is reduced, thereby lowering placental oxygen tension. It is widely believed that placental hypoxia plays a causal role in the disease process.

The highly conserved hypoxia-inducible family (HIF) of transcription factors is a major player in the physiological response to chronic and acute hypoxia [4]. The HIF family consists of heterodimers comprised of one of three alpha subunits (HIF-1α, HIF-2α and HIF-3α) and a beta subunit (HIF-1β). Under hypoxic conditions the alpha subunits are stable, allowing it to accumulate in the nucleus, where upon binding to HIF-1β it recognises HIF-responsive elements (HRE) within the promoter regions of hypoxia-responsive target genes. Under normoxic conditions, the alpha subunits are rapidly degraded by means of ubiquitination and proteasomal degradation [5], [6], [7], [8]. The ubiquitination process requires the product of the von Hippel-Lindau tumor suppressor gene (*VHL*), which functions as a substrate recognition component of an E3 ubiquitin ligase complex [5], [6], [7], [8]. The most extensively studied isoform of the α-subunits is HIF-1α. Oxygen-dependent prolyl hydroxylases control the abundance of HIF-1α by hydroxylating two specific proline residues (402 and 564), an event which is required for VHL binding and subsequent HIF-1α degradation [9], [10]. The prolyl hydroxylase-domain containing proteins 1, 2 and 3 (PHD1, PHD2 and PHD3) function as oxygen sensors as they require O_2_ as co-substrate to catalyze the prolyl-hydroxylation reaction, indicating that oxygen levels directly influence their enzymatic activity [11], [12], [13]. Moreover, *in vitro* experiments have shown that PHDs mRNA levels are up-regulated in conditions of low oxygen [14], further highlighting their role as O_2_ sensors. In contrast to HIF-1α, the stability of PHD1 and PHD3 decreases under hypoxic conditions [15]. Recent studies have shown that under hypoxic conditions, PHD1 and 3 are degraded by specific E3-ubiquitin-ligases, termed SIAHs [Seven In Absentia Homologues] [15], [16]. There are two known human SIAH genes, SIAH-1 (that encodes for two different isoforms: SIAH-1a and SIAH-1b) and SIAH-2. Like PHDs, hypoxia stimulates their transcription and induces the accumulation of these ring finger proteins through an HIF-independent manner [15]. Under hypoxic conditions, SIAHs promote degradation of PHD1 and PHD3 [15], [16], leading to an increased accumulation of HIF-1α, whereas under normoxic conditions PHDs are stable and hydroxylate HIF-1α to target it for degradation [9], [10].

Another oxygen-dependent mechanism of HIF-1α regulation involves the Factor Inhibiting HIF (FIH), an asparginyl hydroxylase that targets the Asn803 residue in the C-TAD domain for hydroxylation. This post-translational modification prevents C-TAD binding to the transcriptional activator p300/CBP, thereby repressing HIF-1α transcriptional activity [17], [18]. Like PHDs, FIH has also been characterized as an oxygen sensor since its enzymatic activity is directly regulated by O_2_ concentration [19].

A number of *in vitro* and *in vivo* studies have highlighted the importance of HIF-1 in placental development and function [20], [21], [22], [23], and, more recently, the regulation of HIF-1α activity and degradation [24]. We and others have reported that HIF-1α levels are increased in preeclamptic placentae [25], [26], but the precise underlying mechanism for this increase in HIF-1α expression remains unknown. Herein, we examined whether dysregulation of the oxygen sensing mechanism and consequently, HIF-1α stability, may be responsible for the increased HIF-1α levels in preeclampsia. In particular, we investigated the expression of oxygen-dependent PHDs, SIAHs and FIH in preeclamptic tissues to determine whether or not the preeclamptic placenta is able to properly sense oxygen tension variations thereby regulating HIF-1α stability and activity.

## Materials and Methods

### Ethics Statement

This study was conducted according to the principles expressed in the Declaration of Helsinki. The study was approved by the Institutional Review Board of Mount Sinai Hospital. All patients provided written informed consent for the collection of samples and subsequent analysis.

### Tissue Collection

First and second trimester human placental tissues (6–15 weeks' gestation, n = 18) were obtained from elective terminations of pregnancies. Seventy six placentae were collected from pregnancies complicated by preeclampsia (PE). The diagnosis of PE was made according to the following criteria: presence of pregnancy-induced hypertension (systolic ≥140 mmHg, diastolic ≥90 mmHg) and proteinuria (≥300 mg/24 h) after the 20^th^ weeks of gestation in normotensive women [2]. Differential diagnosis of early-severe preeclampsia and late-onset preeclampsia was made according to the ACOG criteria [2]. Fifty-eight age-matched control placentae were obtained from normal pregnancies that did not show any signs of preeclampsia or other placental disease. Patients with diabetes, infections and kidney disease were excluded. Clinical data are summarized in Table 1. Maternal age, gestational age and parity were comparable between E-PE *vs* preterm controls (PTC) and L-PE *vs* term controls (TC) groups. Ethnical origins were similar among the four study groups. Only 19.6% of the babies from early-onset preeclamptic pregnancies were growth restricted. Samples were collected randomly from central and peripheral placental areas and snap frozen immediately after delivery. Calcified, necrotic and visually ischemic areas were excluded from collection. Analysis for mRNA and protein were performed in the same samples.

**Table 1:** Clinical Parameters of Control and Preeclamptic Participants.

	Pre-Term Controls	Early Onset Preeclampsia	Term Controls	Late Onset Preeclampsia
	(n = 53)	(n = 58)	(n = 16)	(n = 18)
**Mean Maternal Age (yr)**	33.4±4.8	29.9±5.2	29±4.6	28±4.2
**G.A. at delivery[wk (range)]**	29±3.3 (25–35)	28.6±2.86 (24–35)	39.6±0.6 (39–41)	38±1.5 (36–41)
**Parity**	0.6±0.7	0.63±1.1	0.2±0.4	0.33±0.57
**Blood Pressure (mmHg)**	Systolic: 112±7.5	Systolic: 175.5±16	Systolic: 110.6±6.5	Systolic: 155±11
	Diastolic: 68.7±5.7	Diastolic: 108±14.4	Diastolic: 67.3±6.9	Diastolic: 96±4.2
**Proteinuria (g/24 h)**	Absent	3.0 ±1	Absent	3.0±1
**Fetal Weight (g)**	A.G.A. (n = 53): 1488.2±668.2	A.G.A. (n = 48): 1250±387	A.G.A. (n = 16): 3603±329	A.G.A. (n = 18): 3556.6±588
		IUGR (n = 10): 816.5±394		
**Fetal Sex**	Males: 45%	Males: 66%	Males: 33%	Males: 83%
	Female: 55%	Females: 34%	Females: 67%	Females: 17%
**Mode of delivery**	CS: 41%	CS: 90%	CS: 33%	CS: 71%
	VD: 59%	VD: 10%	VD: 67%	VD: 29%

- Data are represented as mean ± standard deviation.

- G.A.: Gestational Age.

- A.G.A.: Appropriate for Gestational Age.

- IUGR: Intra-Uterine Growth Restriction (<5%).

- VD: Vaginal Delivery.

- CS: Caesarean Section delivery.

### Human Villous Explant Culture

Early (n = 12) and late-onset (n = 3) preeclamptic and age-matched control (n = 8) villous explant cultures were established as previously described [27]. Villous explants were cultured for 4 days under standard tissue culture conditions of 5% CO_2_ in 95% air (20% O_2_ environment) or maintained in an atmosphere of either 3% O_2_/92% N_2_/5% CO_2_. Twenty and 3% O_2_ concentrations were chosen since they represent the standard culturing condition and the physiological placental O_2_ environment before 10 weeks of gestation respectively. Hence, when using third trimester tissue that is physiologically at 5–8% O_2_, oxygen concentration of 3% can be efficiently used to mimic hypoxia. For each treatment, tissue samples from the same placenta were used and in each experiment, explant cultures were set up in triplicate.

### *In-situ* Hybridization

Antisense and sense digoxigenin-labeled HIF-1α riboprobes were generated according to manufacturer's protocol (Boehringer Mannheim, Montreal, QC, Canada). *In situ* hybridization to preeclamptic (n = 5) and normal age-matched control (n = 3) placental tissue sections was performed as previously described [27]. Endogenous alkaline phosphatase was blocked by the addition of 2 mM levamisole. Sections were counter-stained with methyl green.

### FIH silencing

JEG-3 choriocarcinoma cells (ATCC, Manassas, VA, USA) were plated at a density of 2×10^5^ cells/well in 6 well plates and cultured in Eagle's minimal essential medium (EMEM) (ATCC, Manassas, VA, USA) at standard conditions (5% CO_2_ in 95% air). When cells reached 50–70% confluency they were transfected with 30 nM of *Silencer®* siRNA directed against the human FIH gene (Ambion, Inc., Austin, TX, USA) using Lipofectamine™ 2000 (Invitrogen, Carlsbad, CA, USA) following manufacturer's protocol. *Silencer®* Negative Control siRNA (Ambion, Inc., Austin, TX, USA), which does not target any gene product was used as a control.

### RNA isolation and Real Time PCR

Total RNA, extracted from placental tissues and FIH siRNA-treated JEG-3 cells using TRIZOL (Invitrogen Canada Inc, Burlington, ON, Canada), was treated with DNAse I to remove genomic DNA contamination. One µg of total RNA was reverse transcribed using random hexamers (Applied Biosystems (ABI), Foster City, CA, USA). The resulting templates (30 ng of cDNA for our target genes and 1.5 ng for 18S) were quantified by real-time PCR (DNA Engine Opticon2 R system, MJ Research, Waltham, MA). TaqMan probes for PHD1, PHD2, and PHD3 were purchased from ABI. Primers were obtained from the oligosynthesis service at the Hospital for Sick Children (Toronto, Canada). SIAH1, SIAH2, FIH and ribosomal 18S probes and primers were purchased from ABI as Assays-on-Demand™ for human genes. For each probe a dilution series determined the efficiency of amplification of each primer/probe set and the relative quantification method was employed [28]. For the relative quantitation, PCR signals were compared among groups after normalization using 18S as internal reference. Relative expression and fold change was calculated according to Livak and Schmittgen [28].

### Semi- quantitative RT-PCR for SIAH-1b isoform

One µg of total RNA was reverse transcribed using random hexamers (Applied Biosystems). Semi-quantitative PCR was performed using primer sets specific for SIAH-1b (NM 001006610; gi: 55749556): forward primer, 5′-ATGACGGGAAAGGCTACTCCA-3′; reverse primer, 5′-AGTTGCGAATGGATCCCAAA-3′ (predicted amplicon of 346 bp). Human β-Actin (forward primer: 5′-CGAGAAGATGACCCA GATCATGT-3′; reverse primer: 5′-CCACAGGACTCCATGCCCAGGAA-3′) was used as housekeeping gene to normalize the data. DNA contamination was excluded by performing PCR on each sample without first transcribing mRNA with reverse transcriptase.

### Preparation of HIF mutants

The human full-length HIF-1α cDNA construct (generous gift of Dr. Semenza, Johns Hopkins University) was used as template to generate single (HIF-1α_P402R_, HIF-1α_P564R_) and double (HIF-1α_P402R,P564R,P_) HIF-1α mutants using the QuickChange kit (Stratagene, Montreal, QE, Canada). All mutations were confirmed by DNA sequencing.

### Antibodies

Mouse monoclonal antibodies (anti-HIF-1α_P402_^OH^ or anti-HIF-1α_P564_^OH^) were raised against either hydroxylated proline residue 402 or 564 containing peptides of the HIF-1αODD region (Monoclonal Antibody Facility, Hospital for Sick Children).

### Western Blot analysis

Western blot analyses were performed as previously described [25]. Primary antibodies were mouse monoclonal anti-human HIF-1α (1∶250: Affinity Bioreagents Inc., Golden, CO, USA) rabbit polyclonal anti-human PHD1, PHD2 and PHD3 (1∶1000; Novus Biologicals, Littleton, CO, USA), goat polyclonal anti-human SIAH1 and SIAH2 (1∶200 dilution for SIAH1 and 1∶100 dilution for SIAH2; Santa Cruz Biotechnology, Santa Cruz, CA, USA), and FIH (1∶500, Abcam Inc, Cambridge, MA, USA). Horseradish peroxidase–conjugated secondary antibodies were goat anti-mouse for HIF-1α (1∶5000), donkey anti-rabbit for PHDs and FIH (1∶10000) and donkey anti-goat for SIAHs (1∶5000). Specificity of SIAHs antibodies was determined using Siah-1 and Siah-2 blocking-peptides (Santa Cruz Biotechnology). HISM and IL4-treated Ramos cell lysates (Santa Cruz Biotechnology) were used as positive controls for SIAH-1 and SIAH-2, respectively.

### Statistical analysis

All data are represented as mean ± SEM. For comparison of data between multiple groups we used Kruskal-Wallis test and for comparison between two groups we used Mann-Whitney U test. Statistical test were carried out using Prism statistical software and significance was accepted at P<0.05.

## Results

### Oxygen sensing in normal and preeclamptic placentae

**Figure 1:**
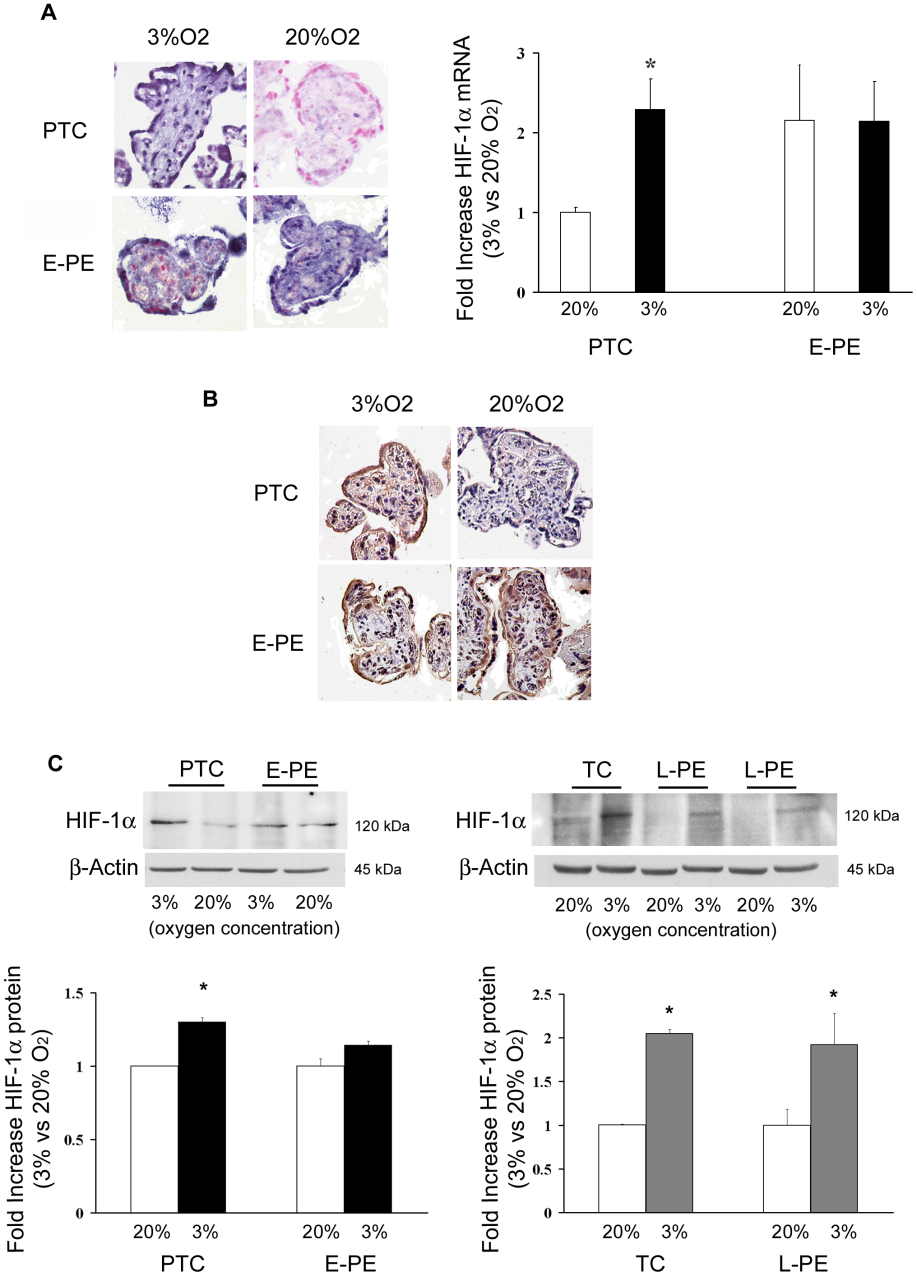
Expression of HIF-1α in preeclamptic and control placental explants. (**A**) Left panel: *In situ* hybridization of HIF-1α mRNA in early preeclamptic (E-PE; n = 5) and preterm control (PTC; n = 3) explants exposed at 3% and 20% O_2_. Blue staining represents positive immunoreactivity for HIF-1α mRNA using a digoxigenin-labeled riboprobe. Right panel: HIF-1α mRNA expression in E-PE (n = 4, black bars) and PTC (n = 3, open bars) explants exposed at 3% and 20% O_2_ as determined by real-time PCR analysis (values are mean ± SEM, *p<0.05). (**B**) Immunohistochemical analysis of HIF-1α protein on sections of E-PE (n = 5) and PTC (n = 3) explants exposed at 3% and 20% O_2_. Brownish staining represents positive immunoreactivity for HIF-1α protein. (**C**) Left panel: Representative HIF-1α immunoblot of E-PE (n = 3) vs PTC (n = 3) (upper panel) and densitometric analysis of HIF-1α protein expression in E-PE (n = 4, black bars) and PTC (n = 3, open bars) explants exposed at 3% and 20% O_2_ (lower panel). (C) Right panel: Representative HIF-1α immunoblot of late preeclamptic (L-PE; n = 3) vs term controls (TC; n = 3,) villous explants cultured at both 3% (open bars) and 20% O_2_ (open bars). β-actin was used as loading control. Data are mean ± SEM, *p<0.05).

We have recently reported striking similarities in global patterns of gene expression between preeclamptic and high altitude placental tissue as well as low oxygen-treated first trimester placental explants [29]. HIF-1α exhibited greater expression in all three low oxygen conditions relative to control [29]. Whether hypoxia or altered oxygen-dependent regulatory mechanisms are responsible for the up-regulated HIF-1α expression in PE placentae is unknown. Therefore, villous explants from early onset (E-PE) and late onset (L-PE) preeclamptic placentae and from age-matched control tissues (pre-term: PTC; term control: TC) were maintained in either 3% or 20% O_2_. The expression and localization of HIF-1α mRNA and protein was determined. *In situ* hybridization (ISH) revealed HIF-1α transcripts in trophoblasts and stroma of PTC control explants cultured at 3% O_2_ (Figure 1A, upper left panel). As anticipated, HIF-1α mRNA was abundant in E-PE explants maintained at 3% O_2_, but in contrast to PTC control explants, HIF-1α transcript levels remained high in E-PE explants cultured at 20% O_2_ (Figure 1A, bottom left panel). No specific staining was observed in control sections hybridized with sense HIF-1α probes (data not shown). Real-time PCR confirmed the ISH data (Figure 1A, right panel). Immunohistochemical (IHC) analysis showed strong positive immunoreactivity for HIF-1α protein in villous trophoblasts of both E-PE and PTC explants maintained at 3% O_2_ (Figure 1B). Low/absent immunoreactivity for HIF-1α was noted in PTC explants cultured at 20% O_2_, while E-PE trophoblasts exhibited strong positive HIF-1α immunoreactivity even when maintained in 20% O_2_. Western blot analysis verified the IHC findings (PTC explants: 3% vs. 20% 1.3-fold increase, p = 0.04; E-PE explants: 3% vs. 20% 1.14-fold increase, ns) (Figure 1C, left panel). These data suggest that E-PE placentae have lost their ability to properly respond to variations in oxygen tension. HIF-1α expression was next examined in placentae from late-onset preeclampsia. As expected, term control explants showed increased HIF-1α protein expression at 3% O_2_ (2.05-fold increase, p<0.01), which was markedly down regulated when the explants were maintained at 20% O_2_ (Figure 1C, right panel). In contrast to E-PE placentae, L-PE explants showed a similar O_2_ response, namely elevated HIF-1α protein at 3% O_2_ (1.92-fld increase, p<0.01) and reduced levels at 20% O_2_.

### Expression of Prolyl Hydroxylases 1, 2 and 3 in normal and preeclamptic placentae

**Figure 2:**
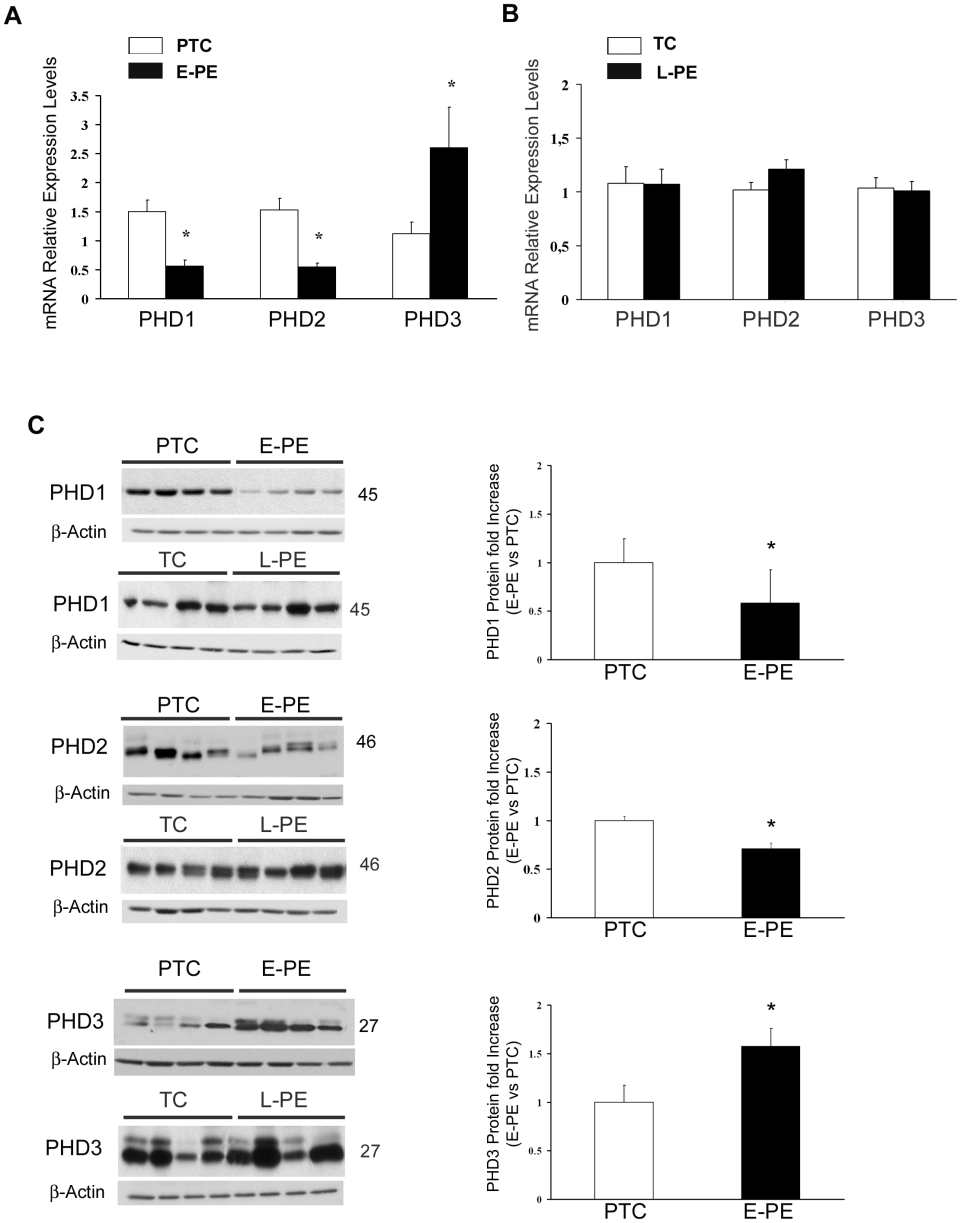
PHDs expression in early preeclamptic (E-PE), late preeclamptic (L-PE), preterm control (PTC) and term control (TC) placentae. (**A**) Expression of PHD1-3 mRNA in E-PE (n = 18) and PTC (n = 15) placentae as assessed by real-time PCR analysis (values are mean ± SEM, *p<0.05). (**B**) Expression of PHD1-3 mRNA in L-PE (n = 12) and TC (n = 10) placentae. (**C**) Left panel: Representative immunoblots for PHDs of E-PE (n = 25), L-PE (n = 11), PTC (n = 19) and TC (n = 8) placental tissues. β-actin was used as loading control. Right panel: Densitometric analysis for PHDs protein levels of E-PE and PTC placentae. Data are mean ± SEM, *p<0.05.

PHDs hydroxylate HIF-1α, thereby targeting it for degradation [11]. In addition, as they utilize molecular oxygen to elicit their function, they have been shown to function as oxygen sensors in a variety of systems [13], [14], including the human placenta [24]. Since E-PE explants showed a lack of oxygen sensing with respect to HIF-1α expression, we next investigated the expression of PHD1, PHD2 and PHD3 in early (E-PE) and late (L-PE) onset preeclamptic placentae. Real-time PCR analysis showed that PHD1 and PHD2, mRNA expressions were decreased in placentae from early preeclamptic pregnancies compared to pre-term controls (Figure 2A). Similar to the mRNA findings, PHD1 (1.7-fold decrease, p = 0.001) and PHD2 (1.69-fold decrease, p = 0.028) protein content was significantly reduced in E-PE placentae compared to PTC controls (Figure 2C). Notably, both PHD3 mRNA and protein expression levels were significantly increased in E-PE placentae relative to controls (Figure 2A, and 2C). Neither mRNA nor protein expression of any PHDs was altered in placentae from pregnancies complicated by late-onset preeclampsia (L-PE) compared to term-control (TC) placentae (Figure 2B and 2C). Because of the high percentage of caesarean section deliveries (CS) in the early- and late-onset preeclamptic population, the expression of PHDs was also examined in normal placentae from CS and spontaneous vaginal deliveries. No differences in PHDs mRNA and protein expression were found between the 2 (control) groups, indicating that changes in PHDs expression do not reflect the mode of delivery (data not shown).

**Figure 3:**
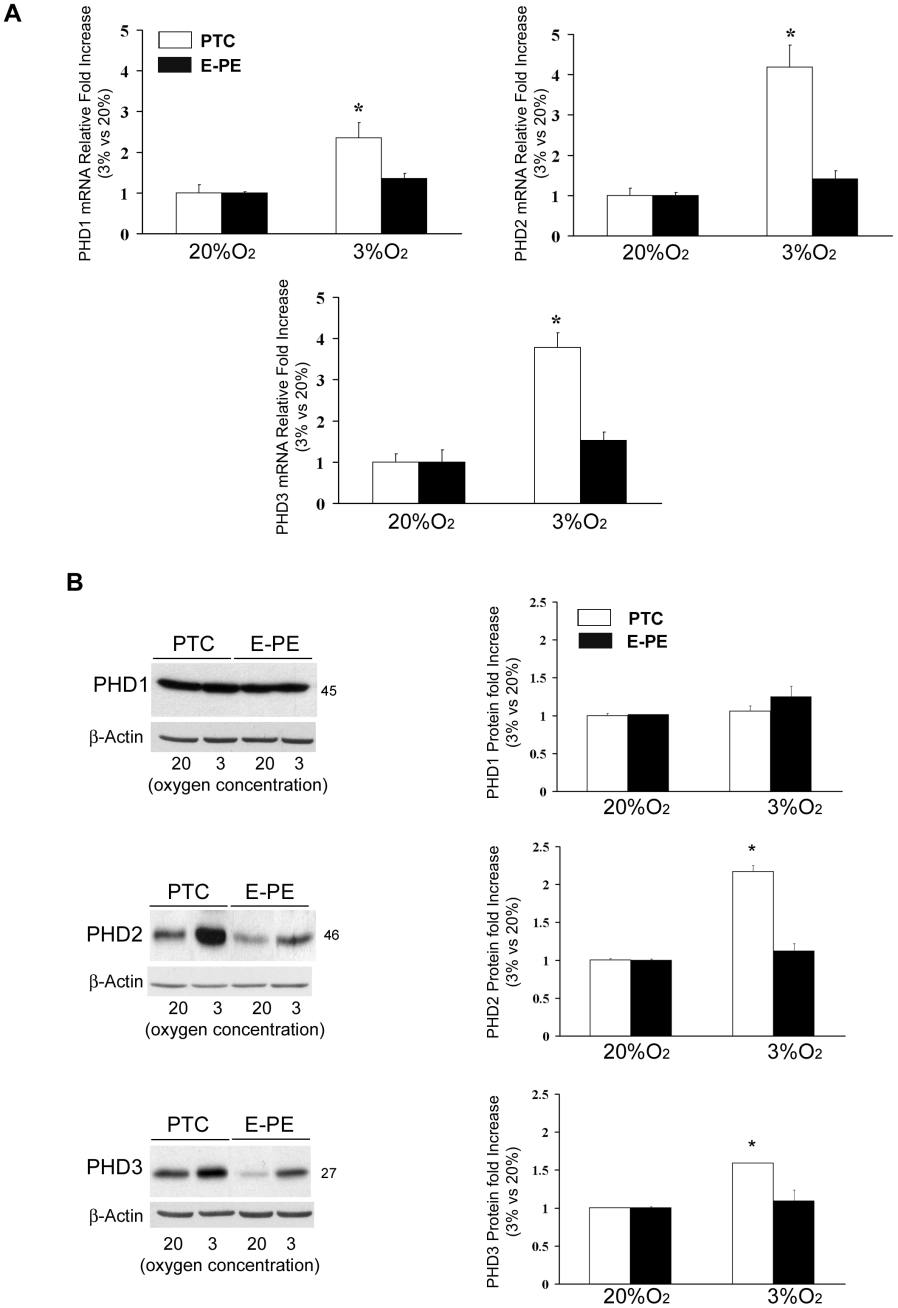
Expression of PHDs in early preeclamptic(E-PE) and preterm Control (PTC) placental explants. (**A**) Expression of PHDs mRNA in E-PE (black bars, n = 4) and PTC (open bars, n = 3) explants exposed at 3% and 20% O_2_ as assessed by real-time PCR analysis (values are mean ± SEM, *p<0.05). (**B**) **Left panel:** Rrepresentative immunoblots for PHDs of E-PE (n = 4) and PTC (n = 3) villous explants cultured at either 3% or 20% oxygen. β-actin was used as loading control. Right panel: Densitometric analysis for PHDs protein levels of E-PE and PTC explants. Data are mean ± SEM, *p<0.05.

Next, we investigated whether the reduction in PHD1 and PHD2 expression in early onset PE was due to a lack of proper oxygen sensing. E-PE and PTC villous explants were cultured at 3% and 20% O_2_ and PHD expression was assessed. Low oxygen (3% O_2_) induced a significant increase in PHD1, PHD2 and PHD3 mRNA expression in normal PTC explants when compared to 20% O_2_ (Figure 3A). The highest induction was observed for PHD2 and 3. No significant oxygen-dependent changes in PHDs mRNA expression were observed in E-PE explants (Figure 3A), suggesting that E-PE placentae fail to sense changes in oxygenation. At the protein level, control explants responded to variations in O_2_ tension by increasing PHD2 (2.17-fold increase, p<0.01) and, to a lesser extent, PHD3 (1.5-fold increase, p<0.05) at 3% pO_2_. E-PE explants showed a modest increase in PHD2 and PHD3 protein expression at 3% pO_2_, while the overall protein levels of PHD2 and 3 were decreased in E-PE when compared to PTC explants. These data confirm the lack of oxygen sensing observed *in vivo.* No changes in response to varying oxygen concentrations were found for PHD1 in both PTC and E-PE explants (Figure 3B).

### HIF-1α hydroxylation during human placental development and in preeclampsia

**Figure 4:**
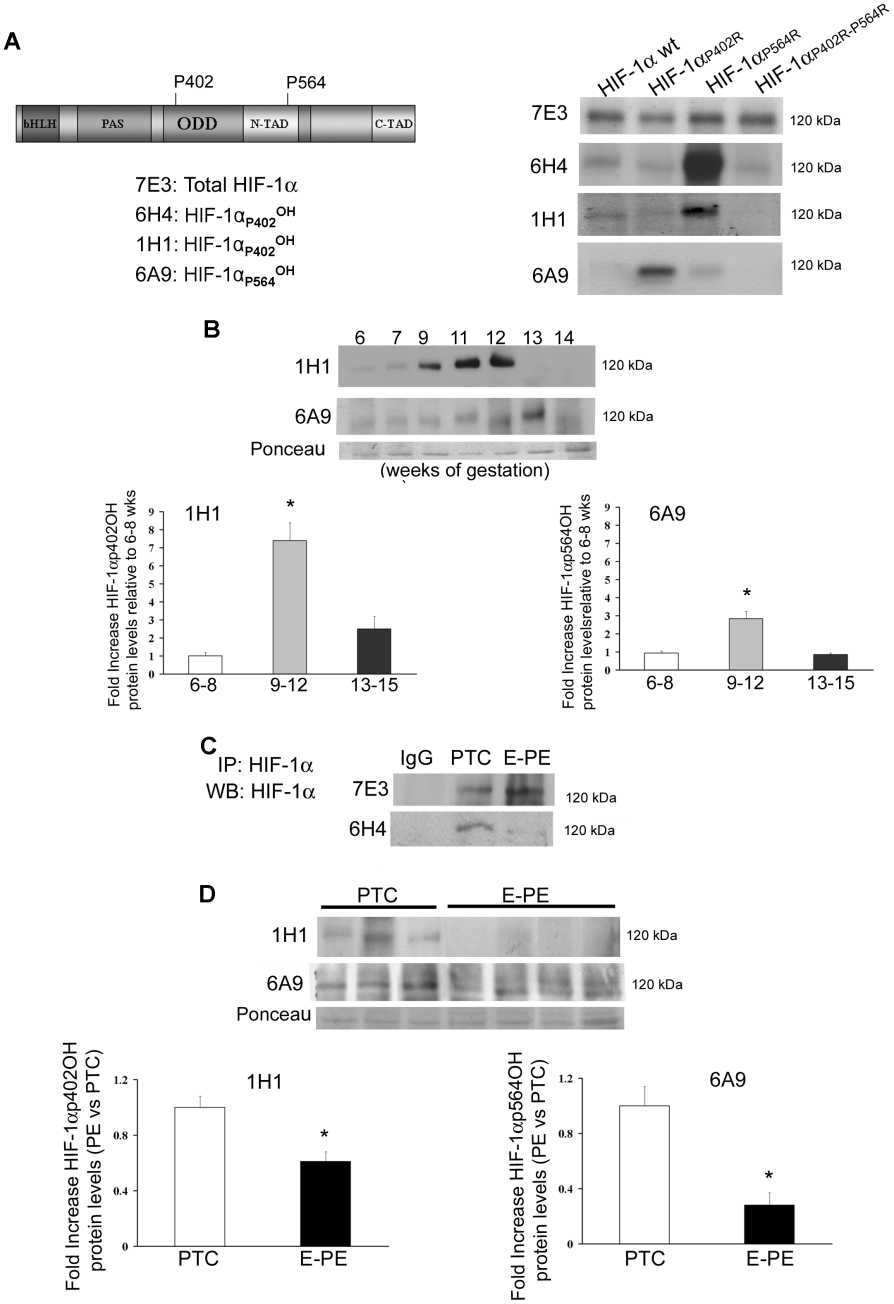
HIF-1α hydroxylation during normal placentation and in preeclampsia. (**A**) Validation of monoclonal antibodies against hydroxylated proline 402 or 564 containing peptides of the HIF-1αODD region. Clones 6H4 and 1H1 recognized specifically HIF-1α hydroxylated at proline 402 as demonstrated by Western blotting of lysates of JEG-3 cells transfected with a HIF-1α single or double mutated construct at P402 and/or P564, while clone 6A9 was specific for hydroxylated proline 564. (**B**) Upper panel: Representative immunoblots for HIF-1α hydroxylated at either proline 402 or 564 during early placental development (6-14 weeks, n = 18), Ponceau staining demonstrated equal protein loading. Lower panel: Densitometric analysis; data are mean ± SEM, *p<0.05.(**C**) HIF-1α immunoprecipitation followed by immunoblotting with monoclonals either recognizing total HIF-1α (7E3) or HIF-1α hydroxylated at proline 402 (6H4). (**D**) Upper panel: Representative immunoblots showing reduced HIF-1α hydroxylation at P402 (1H1) and P564 (6A9) in preeclamptic placentae (E-PE, n = 12) relative to preterm controls (PTC n = 12). Ponceau staining demonstrated equal protein loading. Lower panel: Densitometric analysis of clones recognizing HIF-1α hydroxylation at either P402 (1H1) or P564 (6A9) in preeclamptic placentae vs preterm controls; data are mean ± SEM, *p<0.05.

To establish PHD activities we examined HIF-1α hydroxylation using specific mouse monoclonal antibodies raised against either hydroxylated proline 402 or 564 containing peptides of the HIF-1αODD region. To validate the various clones we generated specific HIF-1α expression constructs including full-length HIF-1α, HIF-1α_P402R_, HIF-1α_P564R_ and HIF-1α_P402R,P564R_. In the latter contructs proline residues 402 or 564 alone or together were mutated to alanine, thereby preventing hydroxylation at those sites. Following transfection with the various HIF-1α constructs, JEG-3 choriocarcinoma cells were cultured for 24 h with proteosomal inhibitor MG-132 to prevent HIF degradation. Subsequent Western blot analysis revealed specificity of clones 6H4 and 1H1, respectively, for HIF-1α hydroxylated at P402 while clone 6A9 was specific for hydroxylated HIF-1α at P564 (Figure 4A). Immunoblotting of placental lysates from first trimester gestation (6–14 weeks of gestation, n = 18) showed increased HIF-1α hydroxylation at P402 at 9–12 weeks of gestation (7.39-fold increase, p<0.01) and a peak of hydroxylation at P564 at 11–13 (2.83-fold increase, p<0.01) (Figure 4B). In E-PE we found that HIF-1α hydroxylation at proline residue 402 was markedly decreased relative to preterm controls (Figure 4C and 4D) (E-PE vs. PTC, 1H1: 1.40-fold decrease; 6H4: 1.44-fold decrease; *p<0.01). A similar decrease was noted for HIF-1α hydroxylation at proline residue 564 (E-PE vs. PTC, 6A9: 1.72-fold decrease). (Figure 4D).

### Expression of Seven in Absentia Homologues 1 and 2 in normal and preeclamptic placentae

**Figure 5:**
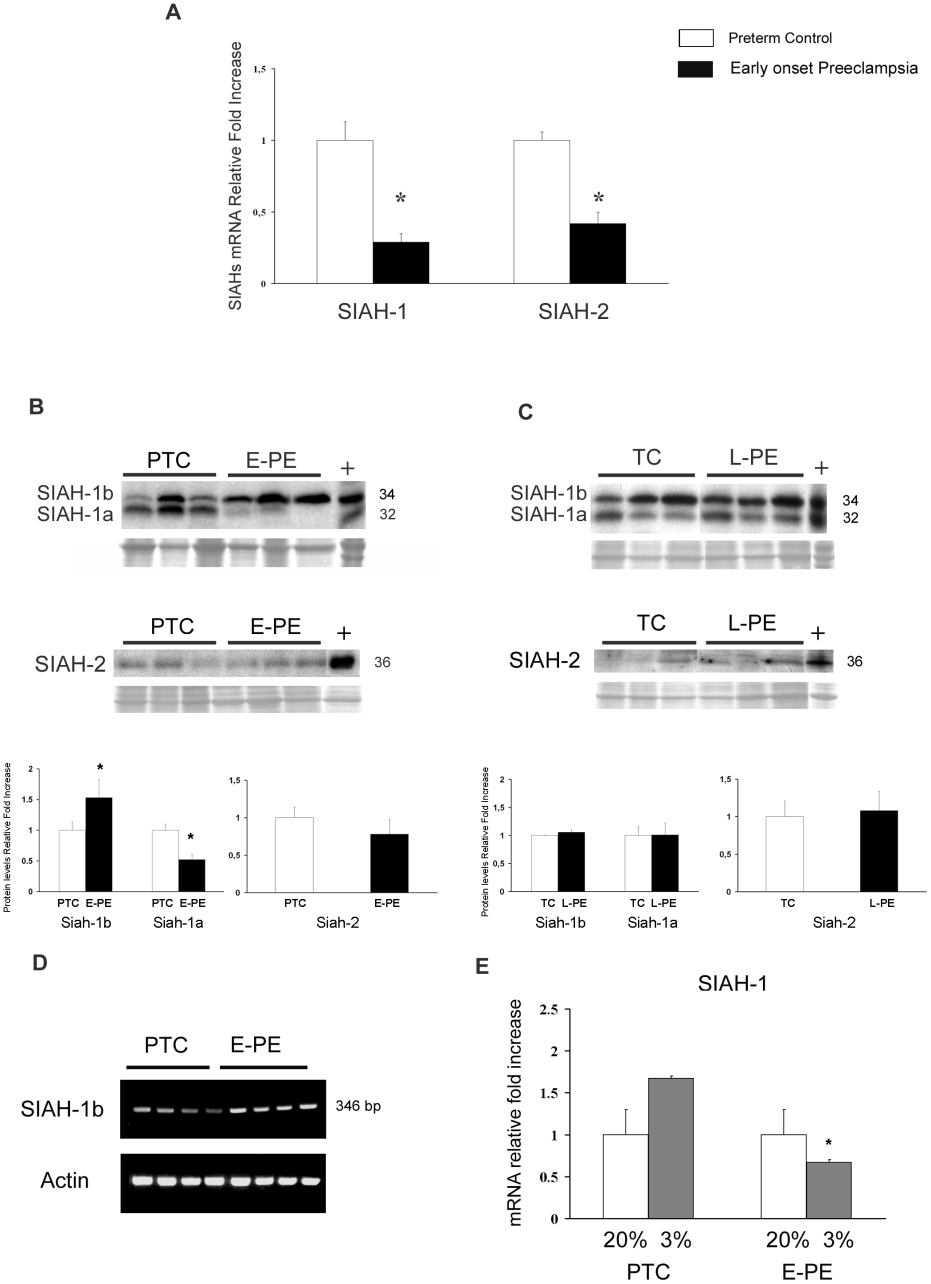
Expression of SIAH-1 and SIAH-2 in early preeclamptic (E-PE), late preeclamptic (L-PE), preterm control (PTC) and term control (TC) placentae. (**A**) Expression of SIAH-1 and SIAH-2 mRNA in E-PE (n = 18, black bars) and PTC (n = 15, open bars) placental tissues as assessed by real-time PCR analysis s (values are mean ± SEM, *p<0.05). (**B**) Upper panel: Representative immunoblots for SIAHs in E-PE (n = 25) and PTC (n = 19) placentae. Ponceau staining demonstrated equal protein loading. Lower panel: Densitometric analysis of SIAHs protein levels in PTC and E-PE placental lysates (data are mean ± SEM, *p<0.05). (**C**) Representative immunoblots for SIAHs in L-PE (n = 12) and TC (n = 10) placentae. Ponceau staining demonstrated equal protein loading. Lower panel: Densitometric analysis of SIAHs protein levels in TC and L-PE placental lysates (data are mean ± SEM, *p<0.05). (**D**) SIAH-1b transcript levels as assessed by semi-quantitative RT-PCR in E-PE (n = 18) vs PTC (n = 15) placentae. Human β-Actin was used as housekeeping gene to normalize the data (lower panel) (**E**) SIAH-1 transcript levels in preeclamptic (n = 4) vs term control placental explants (n = 3) exposed at 3% (gray bars) and 20% (open bars) O_2_; *p<0.05.

The ubiquitin E3 ligases SIAH-1 and SIAH-2 are also oxygen sensors as they are induced by hypoxia [16]. Therefore, we evaluated their expression in placentae from pregnancies complicated by early- and late-onset preeclampsia. Compared to PTC placentae, E-PE placentae showed a significant decrease in both SIAH-1 and SIAH-2 message levels (Figure 5A). Western blot analysis of placental tissues using SIAH-1 antibody revealed the presence of two bands with M_r_ of 32 and 34 kDa (Figure 5B top panel). The SIAH-1 gene is located on chromosome 16q12 and encodes two different isoforms, called SIAH-1a and SIAH-1b, with predicted molecular weights of 32 kDa and 34 kDa, respectively. To demonstrate that the two protein bands represented the two SIAH1 isoforms, we incubated the antibody with competing SIAH-1 peptide prior to Western blotting. Both bands disappeared in positive control and placental tissues, confirming that the antibody recognizes both SIAH-1 isoforms (data not shown). Western blot analysis showed differential changes in SIAH-1a and SIAH-1b protein content between E-PE and PTC tissues (Figure 5B). SIAH-1a protein content was significantly decreased in E-PE placentae relative to preterm controls (1.89-fold decrease, p = 0.004), while SIAH-1b protein showed a significant increase in E-PE *vs.* PTC controls (1.52-fold increase p<0.05). No differences were found in SIAH-2 protein (36 kDa) content between the two groups (Figure 5B bottom panel). In contrast to early-onset PE, late-onset PE did not exhibit an altered protein expression of either SIAH-1 or SIAH-2 (Figure 5C). To determine whether the changes in SIAH-1a and SIAH-1b protein expression between E-PE and PTC placentae were due to altered mRNA expression, we performed semi-quantitative RT-PCR analysis using specific primers for SIAH-1b. Transcript levels of SIAH-1b increased in E-PE placentae in comparison to PTC controls, reflecting the protein pattern (Figure 5D). Hence, the observed reduction in total SIAH-1 mRNA expression (Figure 5A) is likely due to a decrease in SIAH-1a expression.

As for PHDs, we then investigated if the overall reduction of SIAHs was due to a lack of proper oxygen sensing in early-onset preeclamptic placentae. As described earlier, we used E-PE and PTC villous explants which were cultured at 3% and 20% O_2_ and quantified SIAH-1 and -2 mRNA expression levels by real-time PCR. Low oxygen increased SIAH-1 mRNA expression in control explants (Figure 5E). A non-significant increase in SIAH-2 message was noted (data not shown). In contrast, mRNA expression of SIAH-1 (Figure 5E) and SIAH-2 (data not shown) was decreased in E-PE explants cultured at 3% O_2_ compared to explants maintained at 20% O_2_, confirming the *in vivo* findings.

### Expression of Factor Inhibiting Hif-1 in normal and preeclamptic placentae

**Figure 6:**
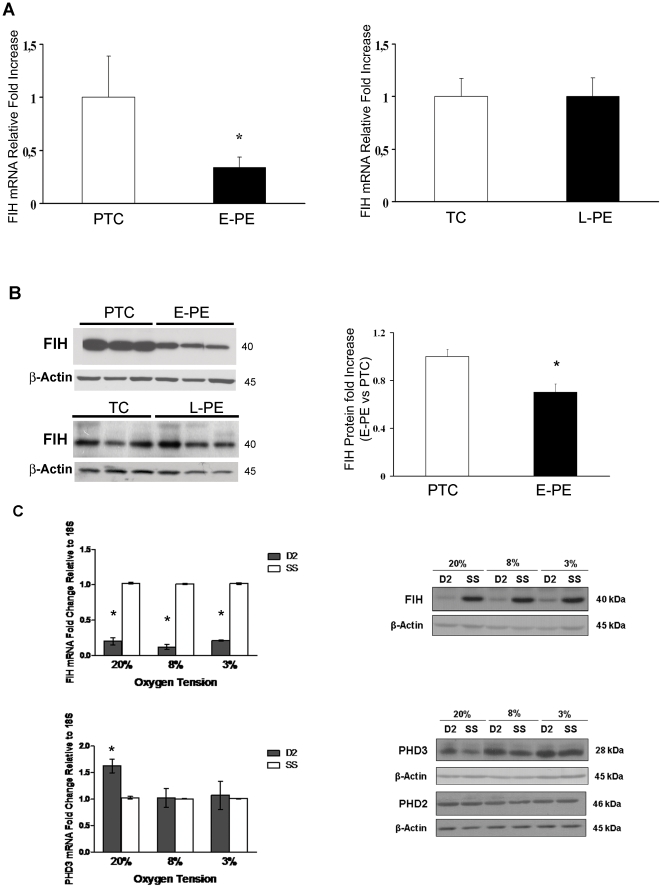
Expression of Factor Inhibiting HIF (FIH) in Preeclamptic Placentae and Pre-term control placentae. (**A**) Expression of FIH mRNA in early onset preeclamptic (E-PE, n = 18) vs pre-term control (PTC, n = 15) placentae (left panel) and late onset preeclmaptic (L-PE, n = 12) vs term control (TC, n = 10) placentae (right panel) as assessed by real-time PCR analysis (values are mean ± SEM, *p<0.05). (**B**) **Left panel:** Representative FIH immunoblots in E-PE (n = 25) vs PTC (n = 19) placentae (left panel) and L-PE (n = 11) vs TC (n = 8) placentae (right panel). β-actin was used as loading control. Right panel: Densitometric analysis of FIH protein expression in E-PE vs. PTC placentae (data are mean ± SEM, *p<0.05). (**C**) Upper panels: FIH mRNA and protein expression levels in FIH siRNA-treated JEG-3 cells maintained at 20, 8 and 3% O_2_. Lower panels: PHD2 and PHD3 expression in JEG-3 cells treated with FIH siRNA. Data are mean ± SEM, *p<0.05. β-actin was used as loading control.

The asparginyl hydroxylase FIH regulates HIF-1α by repressing its transcriptional activity [17], [18]. FIH enzymatic activity is directly influenced by oxygen concentration within the cell, making FIH an oxygen sensing molecule [19]. Herein, we observed that FIH gene expression was significantly decreased (2.9-fold decrease, p = 0.0005) in E-PE placentae relative to preterm controls (Figure 5A left panel), while, as observed before for PHDs and SIAHs, FIH mRNA expression in L-PE placentae was not different from controls (Figure 6A right panel). These results were confirmed at the protein level. FIH protein levels were markedly reduced in E-PE placental tissues relative to preterm controls (Figure 6B), while no changes were observed in L-PE placentae vs. term controls (Figure 6B).

### FIH Regulation of PHD3 expression in JEG-3 Choriocarcinoma cells

In order to determine the functional significance of FIH in regulating PHD3 expression in the placenta, we examined the consequences of inhibiting FIH on PHD3 expression by employing siRNA technology. As FIH has been shown to regulate PHD3 in an oxygen-dependent manner [30], siRNA experiments were conducted at 20%, 8%, and 3% O_2_ to assess the effect of FIH along an oxygen gradient. Real-time PCR (qRT-PCR) and Western blot analyses demonstrated that FIH expression was significantly silenced at all oxygen tensions tested, at the level of both mRNA (20% O_2_: 0.20±0.05-fold, p<0.05; 8% O_2_: 0.12±0.04-fold, p<0.05; 3% O_2_: 0.21±0.01-fold, p<0.05) and protein (20% O_2_: 0.20±0.05-fold, p<0.05; 8% O_2_: 0.17±0.08-fold, p<0.05; 3% O_2_: 0.32±0.11, p<0.05), relative to their respective scramble sequence controls (SS) (Figure 6C upper panels). Decreased FIH levels were associated with a statistical significant increase in PHD3 mRNA and protein (although not significant) expression at 20% O_2_ (1.63±0.13-fold, p<0.05), but not at 8 and 3% O_2_ (Figure 6C, bottom panels). To confirm that FIH selectively regulates PHD3 in JEG-3 cells, we further examined the expression of PHD2, also a HIF-1 target hydroxylase. As anticipated, PHD2 expression was not affected by FIH silencing, providing further support that PHD3 is selectively subjected to FIH-mediated HIF-1 inactivation in JEG-3 cells (Figure 6C).

## Discussion

In the present study we report a disruption of oxygen sensing in early-onset, but not late-onset, preeclamptic placentae. In E-PE placentae we found decreased expression of PHD1, PHD2, SIAHs and FIH, molecules that are known to be up-regulated in response to low oxygen tensions and to be key regulators of HIF-1α, the major player in the cellular response to hypoxia. The diminished expression and function of these oxygen-sensing molecules contributes to decreased HIF-1α hydroxylation and breakdown, leading to its accumulation in early-onset preeclamptic placenta, thereby affecting the expression of molecules that orchestrate proper trophoblast cell differentiation/invasion [25], [26], [31], [32].

Other classical oxygen sensors such as NADPH oxidase and Heme-oxygenase (HO) have also been found to be disrupted in placentae from pregnancies complicated by preeclampsia. Nox1, a gene encoding a novel NADPH oxidase isoform, is expressed in a variety of placental cells [33] and its protein expression has been reported to be increased in placental syncytium and villous endothelial cells from preeclamptic patients [33]. The expression of the HO-2, one of the three isoenzymes that compose HO, has been found to be reduced in the villous endothelial cells of PE placentae, while no differences were found in trophoblast cells [34]. Together these data suggest that impaired oxygen sensing is an important feature of preeclampsia.

PHDs are key regulators of HIF-1α stability [9], [11]. PHDs are also oxygen-sensing molecules as they respond to low pO_2_ by up-regulating their transcript levels, as demonstrated in several systems, including the human placenta [11], [24]. In HeLa cells, both PHD2 and 3, but not PHD1, transcripts are induced by hypoxia [11]. In the human placenta, we reported that exposure of first trimester placental explants to low pO_2_ resulted in an increase in PHD2 and PHD3 transcripts [24]. Our finding of decreased PHD2 mRNA expression suggests that in E-PE placenta PHD2 is not sensing the low oxygenated environment.

It has been reported that in normoxic conditions only PHD2 controls HIF-1α stability, while PHD1 and 3 do not contribute to HIF-1α hydroxylation [14]. Thus, the decreased PHD2 protein levels in E-PE placentae likely leads to the reported increase in HIF-1α [25], [26]. Supporting this concept are recent findings obtained with PHD knockout mice [35]. Disruption of the PHD2, but not PHD1 and 3, gene led to embryonic lethality and caused placental HIF1-α over-expression that was associated with placental defects such as diminished villous branching [35]. HIF-1α was not up-regulated in the embryonic heart of PHD2 mutant mouse [35], suggesting a specific role for PHD2 as a regulator of HIF-1α stability in murine placenta. Since early placental development occurs in a hypoxic environment [36], [37], the PHD2 knockout results imply an active role of PHD2 during early development, although it has been thought that PHD2 is not functional during hypoxia [38]. Interestingly, the PHD2^−\−^ mouse placenta exhibited significantly decreased levels of GCM1, a molecule implicated in placental branching morphogenesis [39]. GCM1 has been found to be decreased in placenta from pregnancies complicated by preeclampsia [40]. The influence of PHD2 on GCM1 expression further emphasises the importance of this HIF specific prolyl hydroxylase in human placental development and disease.

PHD3 also controls the stability of the HIF alpha subunit [11], [12], although it is more specific for HIF-2α than HIF-1α [41]. Moreover, while PHD2 is the primary enzyme that affects HIF-1α hydroxylation in normoxia, PHD3 seems to be partially active even in conditions of low pO_2_, thereby controlling HIF-1α levels during hypoxia [16]. In the present study, we observed increased PHD3 expression levels in E-PE placentae relative to controls. This finding together with the increased HIF-1α expression in E-PE suggests that this prolyl hydroxylase does not compensate for the reduction in PHD2 expression and further underscores the low affinity of PHD3 for HIF-1α [41].

Reduced PHD expression is generally indicative of reduced activity, but so far no direct or indirect examination of PHDs function in placenta has been reported. Using specific monoclonal antibodies against HIF-1α hydroxylated at residue P402 or P564, we show for the first time that during placental development HIF-1α hydroxylation is maximal at 9-12 weeks of gestation. These data agree with our previously published observation of increased PHDs and decreased HIF1α expression at this specific window of gestation when trophoblast cells experience a rapid increased in oxygenation. Of clinical significance, we found that HIF-1α hydroxylation is markedly reduced in preeclampsia. Since PHD2 appears to be the primary regulator of HIF-1α stability by hydroxylating P402 and 564, it is plausible that the decreased amount of PHD2 protein and activity in E-PE is the main cause of aberrant HIF-1α expression.

Since PHD2 and 3 are induced by low O_2_ via HIF-1 [42], it was surprising that in E-PE placentae the hypoxic environment [29] together with its elevated HIF-1α expression [25], [26], [31], [32] was associated with high levels of PHD3 and not PHD2. However, studies using either pVHL-deficient cells, which have high HIF-1α levels due to fact that HIF-1α is not degraded, or cell lines over-expressing HIF-1α, have shown increases in expression of PHD3 but not PHD2 [43]. Hence, increases in HIF-1α alone are not sufficient to induce PHD2 expression in E-PE placentae. A recent study reports that TGFβ1 negatively regulates PHD2 gene expression [44]. In preliminary experiments, we found that exposure of human villous explants to both TGFβ1 and TGFβ_3_ results in decreased PHD2 mRNA expression. Moreover, TGFβ3 [22] levels are increased in severe preeclamptic placentae and this may explain the reduced PHD2 levels found in this pathology.

While PHD2 and PHD3 are induced by hypoxia via a mechanism that involves HIF-1α PHD1 appears not to be an HIF-1 target gene [11], [41] and may even be inhibited by hypoxia [45]. Other studies have reported that PHD1 expression is regulated by estrogen [41], [45] and it is plausible that the decreased PHD1 expression in preeclampsia is due to an alteration in the hormonal milieu. In rats with hypoxia-induced hypertension PHD1 expression negatively correlated with HIF-3α, but not HIF-1α, expression, suggesting that PHD1 has a greater specificity for HIF-3α [46]. Thus, the low expression of PHD1 in E-PE placentae does likely not contribute to the increase in HIF-1α levels in E-PE.

SIAHs have recently been characterized as novel oxygen-sensing molecules as hypoxia stimulates their transcription and accumulation albeit in a HIF-independent manner [16]. Here, we show for the first time the expression of both SIAH-1 and SIAH-2 in the human placenta. In particular, we found decreased SIAH-1 and SIAH-2 mRNA levels in early-onset, but not late-onset, preeclamptic placentae, further emphasising the lack of placental oxygen sensing in the most severe form of preeclampsia. Recently, a novel splicing variant of SIAH-1, called SIAH-1L, has been reported [47]. This variant corresponds to the placental SIAH-1b isoform that we found in the present study. SIAH-1L has been demonstrated to be induced by p53 and to enhance the degradation of β-catenin, thereby promoting cell apoptosis [47]. Thus, the observed increase in SIAH-1b protein in E-PE placentae may contribute to the increased placental apoptosis seen in preeclampsia [48]. Both SIAH-1 and SIAH-2 mRNA levels were decreased in E-PE, supporting abnormal oxygen sensing, but only SIAH-1a protein was reduced while no changes in SIAH-2 were found in E-PE compared to control placentae. It has been reported that both SIAH-1 and SIAH-2 decrease the abundance of PHD1 and PHD3 [16] and that SIAH-2 is more efficient in degrading PHD3 than PHD1 [49] implicating other proteolytic pathways in regulating PHD1 stability [50].

In conditions of hypoxia PHD3 forms hetero-complexes with PHD2, thereby reducing PHD2 ability to hydroxylate HIF-1α and enhancing its degradation by SIAH-1 and 2 [49]. In preliminary experiments, we found that PHD3 dimerizes with PHD2 in both normal and pathological placental tissues (data not shown). Thus, the normal SIAH-2 and high PHD3 protein levels found in E-PE placentae likely contribute to the reduced PHD2 levels and increased amount of HIF-1α.

Another important level of HIF-1α regulation involves FIH [17], [18], [19]. Like PHDs, FIH is an oxygen-dependent molecule as its enzymatic activity is directly influenced by pO_2_ within the cell [19], [51]. It has been reported that PHDs and FIH have different K_m_ for oxygen [51]. Since these O_2_-dependent molecules act on different HIF-1α domains, an interesting O_2_ regulatory model for HIF-1α activity has been proposed [30]. Along a decreasing gradient of O_2_ tension PHDs are the first sensors to be inactivated, leading to stabilization of HIF-1α and activation of HIF-1α N-TAD transcriptional activity, followed by inhibition of FIH at severe hypoxia, which will lead to the activation of HIF-1α C-TAD transcriptional activity [30]. FIH controls the expression of a variety of genes via its action on C-TAD domain [30] that can be divided in FIH-inhibited and non-FIH-inhibited genes. Vascular endothelial growth factor (VEGF), which expression has been reported to be increased preeclamptic placentae [29], [52], [53], [54], [55] belongs to the FIH-inhibited genes [30]. PHD3 is also inhibited by FIH [30]. In the present study, we observed a dramatic decrease of FIH expression in E-PE placentae, which could explain the increased PHD3 levels found in this pathology. In support of our *in vivo* data we found that FIH silencing in trophoblastic JEG-3 cells increased PHD3, but not PHD2, expression at 20%, but not 3%, O_2_. This discrepancy with the hypoxic E-PE is likely due to the complex *in vivo* placental model versus the simpler JEG-3 system. Recently, we demonstrated that FIH expression is increased in high altitude placentae, a unique physiological model of adaptation to chronic hypoxia [56]. Thus, the low FIH mRNA levels in E-PE placentae accentuate the inability of these placentae to properly sense O_2_. Reduced amounts of FIH protein in combination with increased HIF-1α levels in E-PE placentae probably contributes to the increased VEGF levels previously reported in preeclamptic placentae [29].

Interestingly, we observed normal gene and protein expression of PHDs, SIAHs and FIH in late-onset preeclamptic placentae relative to controls. In stark contrast with E-PE explants, L-PE placentae showed a normal regulation of HIF-1α levels, with higher expression at 3% O_2_ and down-regulation at 20% O_2_. Redman et al. [57] theorized on the basis of clinical features that there are two main categories of preeclampsia, placental (early PE) and maternal (late PE). Our data provide the first molecular evidence for this theory. In preeclampsia of placental origin (E-PE), we demonstrated that PHDs, SIAHs and FIH are unable to properly sense and respond to hypoxia. The consequence is aberrant HIF-1α over-expression typical of E-PE. These features contribute to the morphological, molecular and functional alterations of preeclamptic trophoblasts that characterize early onset preeclampsia as pathology of placental origin. Maternal preeclampsia arises from the interaction between a normal placenta and a maternal constitution that is susceptible to, or suffers from, microvascular disease [57] and as such the late-onset preeclamptic placenta is a system that is able to properly sense and respond to oxygen tension variations.

Our previous findings of placental hypoxia in preeclampsia [27] together with the present data on disruption of oxygen sensing mechanisms explain the elevated HIF-1α levels found in preeclamptic placenta. [25], [26], [31], [32] and the high expression of known HIF-1 targets such as VEGF [29], [52], [53], [54], [55] and sFlt-1 [58], [59], [60], [61]. However, it is possible that the defect in oxygen sensing in the E-PE placenta is an effect and not the cause of the preeclamptic disease and that the HIF-1α response is triggered by other mechanisms [62], [63]. The notion of placental hypoxia as leading cause of placental pathologies is disputed. Others have suggested hypoxia/reoxygenation [64], [65] or hyperoxia as etiological factors in preeclampsia.[66], [67], [68]. Both conditions lead to the generation of reactive oxygen species (ROS) [64], [65] and mitochondrial ROS have been shown to increase HIF-1α levels [69], [70], [71] and to alter expression of PHDs [72] although the findings are controversial as the ROS effect appears to vary between different biological systems. ROS generation is also high during hypoxia and thus may contribute to HIF-1α stabilization under low-oxygen conditions [69], [70], [71], [73], [74].

In conclusion, our molecular data emphasize the existence of two preeclamptic subtypes, early and late onset, respectively, and further highlight the key role of PHD2 in human placental physiology and pathology. Importantly disruption of the oxygen-sensing machinery may be of diagnostic value. Since HIF-1α is crucial for proper placental development, early detection of aberrant HIF-1α regulatory mechanisms could impact on the differential diagnosis between high risk and low risk pregnancies. This may impact on the disease management during pregnancy and may ultimately be translated into novel therapeutic targets. In fact, in cancer research a variety of therapeutic tools aimed at targeting the HIF pathway are currently being developed, hence increasing HIF hydroxylation in order to prevent its accumulation may reduce its detrimental consequences in placental pathologies.
